# Accuracy of Video Optical See-Through Augmented Reality Surgical System (VOSTARS) to Guide Craniotomy: A Phantom and a Human Cadaver Study

**DOI:** 10.3390/brainsci16101112

**Published:** 2026-10-15

**Authors:** Nicola Montemurro, Marina Carbone, Rosanna Maria Viglialoro, Francesco Simi, Giulia Zari, Sara Condino, Francesco Acerbi

**Affiliations:** 1 Department of Neurosurgery, Azienda Ospedaliero Universitaria Pisana (AOUP), 56100 Pisa, Italy; 2 EndoCAS Interdipartimental Center, University of Pisa, 56100 Pisa, Italy; 3 Department of Information Engineering, University of Pisa, 56100 Pisa, Italy; 4 Department of Translational Research and New Technologies in Medicine and Surgery, University of Pisa, 56100 Pisa, Italy

**Keywords:** augmented reality, head-mounted display, image-guided neuronavigation, cadaver study, craniotomy, preoperative planning, neuronavigation, neurosurgery, surgical planning

## Abstract

Background: Augmented reality (AR) may support neuronavigation by displaying patient-specific anatomical information and surgical plans directly within the surgeon’s view of the operative field. Reliable registration between virtual content and patient anatomy remains a critical requirement. Patient-specific templates offer a non-invasive registration strategy, but positioning errors at the facial support may propagate to the surgical target. Methods: We performed a preclinical feasibility evaluation of the Video and Optical See-Through Augmented Reality Surgical System (VOSTARS), a head-mounted AR platform for surgical navigation, using patient-specific template-based registration. Six craniotomies were planned using CT datasets from two fresh-frozen human cadaver heads and two corresponding cranial phantoms were generated from the same datasets. Visualization accuracy, reported as target visualization error (TVE_3_D), and trajectory-tracing performance were first assessed on the phantoms. The complete AR-guided planning-to-execution workflow was subsequently evaluated on the corresponding cadaveric specimens by assessing containment of the AR-traced craniotomy trajectories within patient-specific inspection windows corresponding to a tolerance of ±1.5 mm. Results: In the phantom experiments, the mean TVE_3_D calculated across five of the six planned craniotomies was 1.64 mm, with individual mean values ranging from 1.33 to 1.91 mm. Craniotomy 1 had a mean TVE_3_D of 6.92 mm; the higher error was attributed to incorrect phantom assembly. Four of the six craniotomy trajectories traced on the phantoms under AR guidance showed complete overlap with the corresponding virtual trajectories. For the other two, trajectory overlap was 62.5% and 67.0%, with a mean deviation from the planned trajectory of 0.79 mm in both cases. In the cadaveric experiments, the complete AR-guided planning-to-execution workflow was completed for all six planned craniotomies. Two of the six AR-traced craniotomy trajectories were completely contained within the inspection windows corresponding to the predefined ±1.5-mm tolerance. Of the remaining four trajectories, two were partially contained, one was minimally contained and one was not contained within the inspection window. Conclusions: This study supports the technical feasibility of a patient-specific AR workflow for craniotomy guidance across anatomically different cranial approaches. In this exploratory preclinical study, VOSTARS enabled the translation of patient-specific preoperative planning directly to the operative field and provided AR guidance during the initial stages of cranial surgery. The cadaveric findings also highlight template fit and positioning as important determinants of workflow performance. Further quantitative and comparative validation is required before clinical implementation.

## 1. Introduction

Accurate localization of intracranial pathology and precise planning of the surgical corridor are fundamental principles of modern neurosurgery. Craniotomy represents the first physical translation of the preoperative surgical plan to the patient and directly influences the subsequent surgical approach. The location and geometry of the craniotomy influence the angle of visualization and the instrument trajectory toward the intracranial target. This is particularly relevant in minimally invasive and anatomically constrained approaches, including skull-base, parasagittal, interhemispheric and keyhole approaches, in which even small deviations from the planned craniotomy may alter the intended surgical trajectory and require intraoperative modification of the craniotomy. Preoperative imaging and three-dimensional surgical planning can support the identification of relevant anatomical landmarks and the patient-specific definition of the craniotomy according to the intended surgical approach. However, accurately reproducing the virtual surgical plan on the patient’s anatomy remains a key technical challenge.

The development of high-resolution neuroimaging and image-guided neuronavigation has substantially improved the ability to localize intracranial targets and plan surgical procedures. Conventional commercial neuronavigation systems use optical or electromagnetic tracking to localize the patient and surgical instruments relative to preoperative imaging. Their accuracy depends on the registration methodology and may be affected by changes in the spatial relationship between the preoperative images and the patient’s anatomy during surgery [1,2,3]. Moreover, navigation information is typically displayed on a separate monitor, requiring the surgeon to repeatedly alternate visual attention between the operative field and the navigation interface [1,2].

Augmented reality (AR) provides an alternative visualization interface by superimposing computer-generated information onto the surgeon’s view of the physical environment [3,4,5]. Preoperative imaging, anatomical models and planned surgical trajectories can therefore be visualized in spatial relationship with the patient while maintaining direct observation of the operative field [6,7,8]. Head-mounted display (HMD)-based AR is particularly relevant in this context because it can provide in situ visualization without requiring the surgeon to rely exclusively on a fixed external display. Potential applications in cranial surgery include guidance during the initial stages of the procedure, such as skin-incision marking and craniotomy positioning.

The potential usefulness of AR guidance depends on the accuracy and stability of the registration between the virtual model and the patient’s anatomy. Additional technical and perceptual limitations include depth-perception errors, restricted field of view, system latency, tracking instability and operator-related variability [9,10,11]. Accordingly, clear and application-specific evaluation methods are required, particularly because the metrics used to assess AR navigation remain heterogeneous and are not always directly comparable across studies [2,12].

A systematic review and meta-analysis by Fick et al. [2] reported a mean target registration error of 2.5 mm for AR neuronavigation, compared with 2.6 mm for conventional infrared neuronavigation, suggesting that AR systems can achieve accuracy within the range reported for established navigation technologies. However, the authors also highlighted substantial heterogeneity in registration methods and accuracy assessment protocols [2]. In our previous experimental study, we evaluated the Video and Optical See-Through Augmented Reality Surgical System (VOSTARS) for guiding complex craniotomies using a patient-specific template-based registration method [13]. The system achieved a mean three-dimensional target visualization error of 1.3 ± 0.6 mm, while 97% of the traced trajectory length remained within 1.5 mm of the planned trajectory. These findings supported the feasibility of using a patient-specific template for rapid planning-to-patient registration and suggested that wearable AR could provide an ergonomic interface for translating preoperative planning into the operative field. Subsequent comparative studies evaluated the VOSTARS platform against conventional infrared and electromagnetic neuronavigation systems [14,15,16,17]. However, the previous craniotomy-guidance evaluation [13] was conducted primarily under controlled experimental conditions and did not assess the complete surgical workflow across multiple anatomically distinct cranial approaches.

This represents a relevant limitation for template-based registration: although dedicated arms position the tracking markers close to the planned craniotomy to support robust tracking, the template retains a relatively constant facial support in the periorbital region. Consequently, positioning or angular errors at the facial interface may propagate through the registration and produce larger deviations at surgical targets farther from the support region.

Although the literature on AR in neurosurgery is expanding, available studies use heterogeneous registration strategies, experimental models and outcome measures [10,12,14,15]. In particular, the robustness of patient-specific template-based registration across different cranial approaches and throughout a complete cadaveric workflow remains insufficiently characterized. To address this gap, we evaluated the VOSTARS workflow across six anatomically distinct cranial approaches, selected to represent different spatial relationships between the facial template support, tracking-marker configuration and surgical target. Patient-specific anatomical models were reconstructed from high-resolution CT datasets acquired from the two cadaveric specimens and used to plan the skin-incision and craniotomy trajectories. The phantom experiments quantitatively assessed target visualization error and AR-guided trajectory-tracing performance. The cadaveric experiments evaluated the feasibility of the complete workflow and the correspondence between the planned and AR-traced craniotomy trajectories using patient-specific inspection templates.

The primary objective was to evaluate the feasibility of the VOSTARS workflow for reproducing patient-specific craniotomy plans on physical cranial anatomy. The secondary objective was to assess visualization accuracy and trajectory-tracing performance under controlled phantom conditions.

## 2. Materials and Methods

### 2.1. Study Design

**Figure 1:**
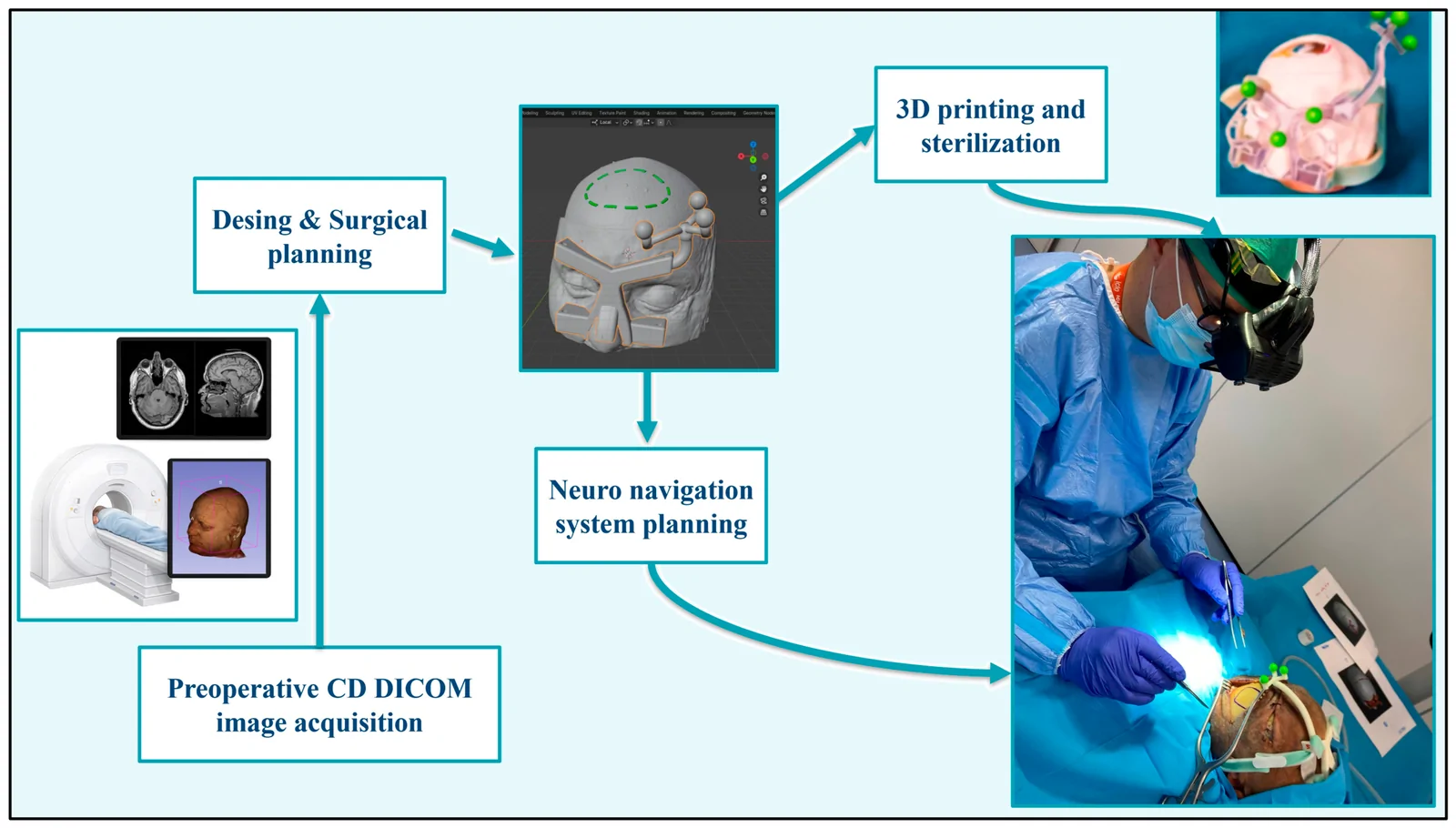
AR-guided neurosurgical workflow. Schematic representation of the workflow from CT acquisition and segmentation through three-dimensional surgical planning, patient-specific template fabrication, virtual-to-real registration, AR-guided surgical marking and experimental assessment.

The study was designed as a two-stage exploratory preclinical technical feasibility study of the VOSTARS HMD-based AR workflow. The workflow was initially evaluated under controlled conditions on patient-specific cranial phantoms and subsequently evaluated on two fresh-frozen human heads. Both experimental stages were based on the same patient-specific CT datasets and included three-dimensional reconstruction, surgical planning, template-based registration and AR-guided marking of the planned trajectories. The overall experimental workflow comprised four sequential steps: (1) acquisition and segmentation of high-resolution CT data; (2) patient-specific 3D reconstruction, surgical planning and design and fabrication of the patient-specific template; (3) template-based registration and AR-guided visualization and marking of the planned trajectories; and (4) experimental assessment of visualization accuracy and AR-guided trajectory transfer (Figure 1). The six craniotomy locations were deliberately distributed across anterior, lateral, paramedian, posterior-fossa and occipital regions to evaluate the same facial-support registration concept across different spatial relationships to the operative target. This exploratory design was intended to reveal potential location-dependent sensitivity to template fit and registration geometry, rather than to establish a formal distance-error model.

### 2.2. Anatomical Specimens and Patient-Specific Reconstruction

For the cadaveric phase, two fresh-frozen human cadaver heads were used. Before the experimental procedures, both specimens underwent high-resolution CT imaging. Images were acquired with an isotropic voxel size of 0.4 × 0.4 × 0.4 mm. The corresponding CT datasets were also used to generate patient-specific anatomical models for use in the phantom experiments. This approach ensured that the physical phantoms and cadaveric specimens were based on the same patient-specific three-dimensional datasets.

The CT DICOM datasets were processed using the semiautomatic EndoCAS segmentation pipeline, based on the open-source software ITK-SNAP 1.5 (www.itksnap.org). The segmentation workflow enabled reconstruction of the relevant anatomical structures, including the cranial bone and external head/skin surface. Manual refinement was performed when required to correct segmentation inaccuracies and ensure adequate anatomical fidelity. The resulting three-dimensional anatomical models were imported into Blender 4.2.1. for visualization and surgical planning. The reconstructed models provided the geometric reference for virtual surgical planning and fabrication of the corresponding physical phantoms.

### 2.3. AR-Guided Surgical Workflow

The VOSTARS platform supports a structured AR-guided workflow that integrates patient-specific three-dimensional surgical planning, template-based virtual-to-real registration and AR-guided surgical marking and execution.

#### 2.3.1. Preoperative Planning and Template Fabrication

Following three-dimensional reconstruction, the anatomical models were imported into Blender, where surgical planning was performed in collaboration with experienced neurosurgeons. Six craniotomy trajectories, corresponding to six different cranial approaches, were planned across the two patient-specific skull models. For each approach, the intended skin incision line and craniotomy trajectory, defining the location and geometry of the planned bone opening, were created according to established neurosurgical approaches and the anatomical characteristics of the individual specimen.

The six planned approaches were: (1) right supraorbital; (2) bifrontal; (3) left temporal; (4) right paramedian pre-rolandic; (5) right retrosigmoid; (6) left paramedian occipital craniotomy. For each planned craniotomy, a patient-specific facial registration template incorporating the tracking markers was designed. The template was generated from the three-dimensional reconstruction of the corresponding head and defined the geometric relationship between the tracking-marker configuration and the patient-specific anatomical model. The templates were fabricated in acrylonitrile butadiene styrene (ABS) using fused deposition modeling (FDM) for experimental testing. Across the six approaches, the patient-contact portion of the template retained its support on the periorbital facial region. The three fluorescent markers were connected to this support by arms designed to position them as close as practicable to the planned craniotomy, while maintaining visibility to VOSTARS and avoiding interference with the surgical workspace. This configuration was intended to preserve favorable tracking conditions; target-level navigation accuracy nevertheless remained dependent on correct and stable placement of the facial support.

The final virtual content, including the patient-specific anatomical models and planned skin-incision and craniotomy trajectories, was expressed in the coordinate system of the corresponding registration template and exported in Virtual Reality Modeling Language (VRML) format for visualization with VOSTARS.

#### 2.3.2. VOSTARS HMD Configuration and Registration

VOSTARS is a hybrid video and optical see-through HMD based on a commercial binocular optical see-through visor (ARS.30, Trivisio SAS, Chantilly, France) equipped with two front-facing RGB cameras (LI-OV4689, Leopard Imaging Inc., Fremont, CA, USA) for AR visualization and tracking. Tracking is based on three fluorescent spherical markers, 12 mm in diameter, integrated into the patient-specific facial template and detected by the cameras incorporated into the HMD. The system architecture and tracking methodology have been described previously [13].

For each craniotomy, the phantom or cadaveric specimen was positioned according to the predefined surgical configuration. The corresponding patient-specific facial template was then placed and securely fixed to the facial surface, with all three markers visible to the HMD cameras. The template served as the physical reference for establishing the spatial relationship between the virtual model and the corresponding physical anatomy. The surgeon then put on the VOSTARS HMD and initiated the model-based virtual-to-real registration procedure. The patient-specific three-dimensional anatomical model was aligned with the corresponding physical anatomy using the template-based registration workflow. Once registration was established, the VOSTARS system displayed the preoperatively planned skin-incision and craniotomy trajectories and anatomical information within the surgeon’s view of the operative field.

#### 2.3.3. AR-Guided Surgical Marking and Execution

In the phantom experiments, the planned craniotomy trajectories were traced directly on the physical phantoms under AR guidance using a marker with a 1-mm tip.

For the cadaveric experiments, the AR-displayed skin incision trajectory was first traced with a sterile dermographic marker with a 1-mm tip, after which the incision was performed with a scalpel. Following exposure of the calvarium, the planned craniotomy was displayed and traced directly on the skull using the same marker. The accuracy of the skin-incision trajectory was not quantitatively assessed. After assessing the traced craniotomy trajectory using the corresponding patient-specific inspection template, as described in Section 2.4.2, the craniotomy was subsequently performed using a Mectron PIEZOSURGERY^®^ Plus (Mectron S.p.A., Carasco, Genoa, Italy) device.

### 2.4. Experimental Evaluation

The VOSTARS workflow was evaluated in two complementary experimental stages: a controlled study on patient-specific cranial phantoms and a subsequent study on two fresh-frozen human cadaver heads. The phantom study assessed TVE and AR-guided trajectory-tracing agreement under controlled conditions. The cadaveric study assessed the feasibility of the complete planning-to-execution workflow and qualitatively evaluated the correspondence between the planned and AR-traced craniotomy trajectories.

#### 2.4.1. Phantom Experiments

Two patient-specific cranial phantoms were fabricated from the corresponding three-dimensional anatomical models in ABS using fused deposition modeling (FDM). Each phantom was covered with a layer of Ecoflex™ silicone rubber (Smooth-On Inc., Macungie, PA, USA), pigmented with Silc Pig™, to simulate the external soft-tissue layer and permit skin incision and flap elevation.

The six planned craniotomy trajectories were distributed across the two phantoms. Trajectories 1 and 3 were performed on Phantom 1, whereas trajectories 2, 4, 5 and 6 were performed on Phantom 2. For each procedure, the phantom was positioned in the predefined surgical configuration using an articulated support arm and the corresponding patient-specific facial registration template was applied before registration. The experimental procedures were recorded using the cameras integrated into the VOSTARS HMD.

For each planned craniotomy, three predefined target points located within the planned craniotomy region were selected to quantitatively evaluate the accuracy of AR visualization. The target points were defined on the preoperative 3D model and corresponded to identifiable physical locations on the phantom. For each craniotomy, three representative video frames were selected. Visualization accuracy was quantified using the Target Visualization Error (TVE), which was calculated for each target point in each selected frame. Initially, TVE was calculated in image space as the two-dimensional distance between the displayed virtual target and the corresponding physical target in the recorded video frames (TVE_2_D, pixels).

To allow comparison in physical units, the pixel measurements were subsequently converted into millimeters (TVE_3_D, mm) using a pixel-to-millimeter scaling factor derived from the known diameter of the tracking markers. The tracking markers had a standardized diameter of 12 mm and therefore provided a reference dimension for conversion from image-space measurements to physical distance. All measurements and subsequent statistical analyses were performed in MATLAB R2022b (MathWorks, Natick, MA, USA). The integrated VOSTARS RGB cameras (Leopard Imaging Inc., Fremont, CA, USA) were used to record experimental procedures, allowing post-hoc analysis of the selected video frames and the spatial relationship between the displayed virtual targets and their corresponding physical locations.

Trajectory-tracing performance was assessed by comparing the line traced by the surgeon with the corresponding virtual craniotomy trajectory displayed by VOSTARS. Trajectory overlap was defined as the percentage of the traced craniotomy line contained within the virtual trajectory. The virtual trajectory was represented as a 2-mm-wide band, while the physical line was traced using a marker with a 1-mm tip. Complete containment of the traced line within the virtual band corresponded to a maximum centerline deviation of 0.5 mm. For each craniotomy, trajectory overlap was calculated on three representative video frames. When the traced line extended beyond the virtual band, the deviation was measured in pixels and converted into millimeters using the marker-based scaling factor.

#### 2.4.2. Human Cadaver Study and Assessment of AR-Guided Trajectory Transfer

**Figure 2:**
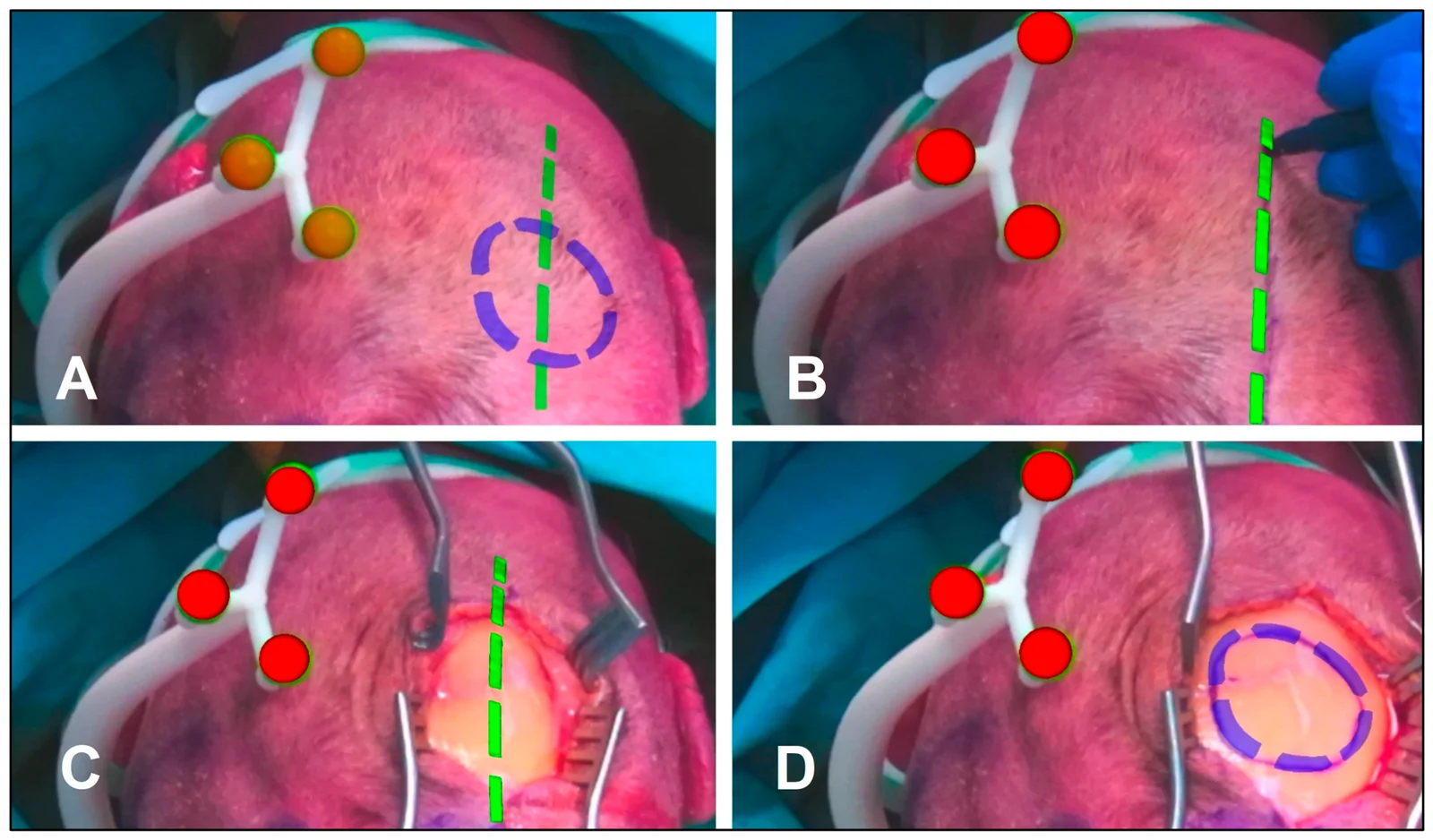
AR-guided cadaveric workflow: visualization of the planned skin-incision (green) and craniotomy (blue) trajectories (**A**), marking of the skin incision under AR guidance (**B**), exposure of the calvarium (**C**) and visualization and marking of the planned craniotomy trajectory on the skull (**D**).

The cadaveric phase was conducted on two fresh-frozen human cadaver heads at the ICLO San Francesco Sales Center (Verona, Italy). The six planned trajectories were distributed between the two specimens following the same allocation used in the phantom experiments: Craniotomies 1 and 3 were performed on Specimen 1, whereas Craniotomies 2, 4, 5 and 6 were performed on Specimen 2. Two neurosurgeons (NM and FA) performed the AR-guided marking and surgical procedures according to the predefined workflow. For each procedure, the patient-specific facial registration template was positioned on the specimen, the VOSTARS system was registered to the anatomical model and the planned skin incision and craniotomy trajectories were superimposed within the surgeon’s view of the operative field. The facial registration template was secured using slightly elastic straps passed through the dedicated template openings and wrapped around the specimen’s head. Supplemental surgical tape was applied when required to improve template stability. After the skin incision and exposure of the cranial vault, the planned craniotomy was marked on the skull and subsequently performed using a piezoelectric device (Figure 2).

**Figure 3:**
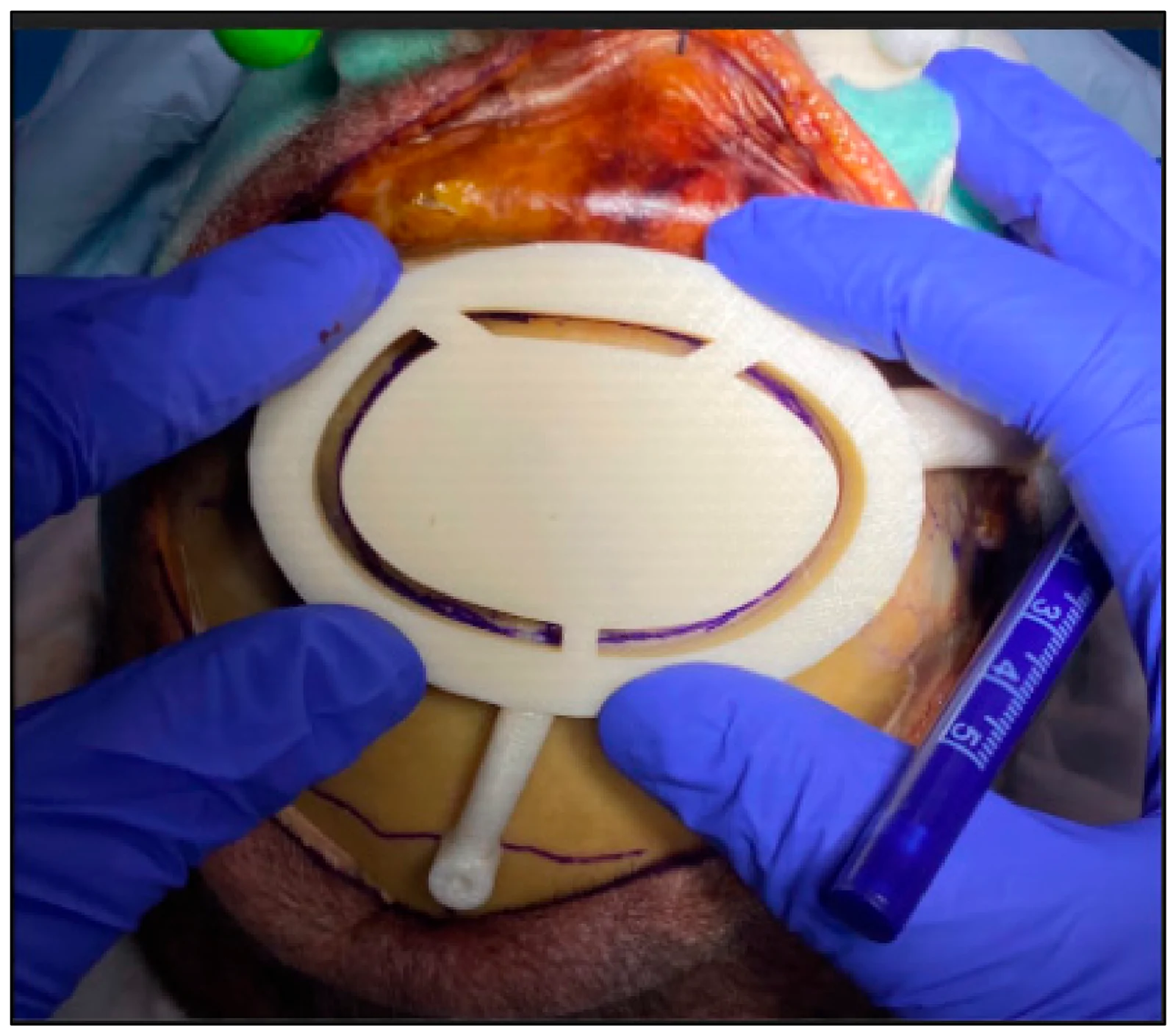
Patient-specific inspection template positioned on the exposed cranial surface using predefined suture landmarks. The 4-mm-wide inspection window was used to assess containment of the AR-traced craniotomy trajectory relative to the planned trajectory.

During preoperative planning, readily identifiable suture-based cranial landmarks were selected on the CT-derived skull models. A separate patient-specific inspection template was designed for each craniotomy. Each template included two positioning arms aligned with the selected landmarks and a 4-mm-wide inspection window reproducing the shape and location of the planned craniotomy trajectory. The inspection templates were fabricated in ABS using fused deposition modeling. Specifically, the intersection of the coronal and sagittal sutures was used in two cases, the intersection of the sagittal and lambdoid sutures in one case, the pterion in two cases and the asterion in one case. After surgical exposure of the cranial surface, the corresponding landmarks were identified on the anatomical specimen and the inspection template was positioned accordingly (Figure 3). After AR-guided marking and before bone cutting, the inspection template was used to assess the correspondence between the traced line and the preoperatively planned craniotomy trajectory. Because the trajectory was traced using a marker with a 1-mm tip, complete containment of the traced line within the 4-mm-wide inspection window corresponded geometrically to a nominal centerline tolerance of ±1.5 mm relative to the planned trajectory. As numerical containment percentages and continuous deviation measurements were not available, trajectory containment was reported descriptively as complete, partial, minimal, or absent. This assessment referred to the AR-traced line before craniotomy execution and not to the geometry of the completed bone opening.

### 2.5. Statistical Analysis

Phantom TVE values were summarized for each craniotomy using the mean, standard deviation and maximum value. Trajectory-tracing performance was reported as the percentage of the traced trajectory contained within the corresponding virtual band and, when incomplete containment occurred, as the mean deviation of the portions extending beyond the band. Because the study was exploratory, each craniotomy was performed once and measurements obtained from multiple targets and video frames within the same procedure were not independent experimental replicates. Accordingly, the analysis was restricted to descriptive statistics, and no inferential statistical comparisons among craniotomies were performed. Craniotomy 1 was reported together with all other procedures in the primary descriptive analysis; an additional descriptive summary of Craniotomies 2–6 was provided because of a documented phantom-assembly problem.

For the cadaveric experiments, results were reported descriptively for each craniotomy. A trajectory was classified as meeting the predefined nominal ±1.5-mm tolerance only when the entire traced line was contained within the corresponding inspection window. Given the limited number of specimens and the single execution of each approach, no inferential statistical analysis was performed.

## 3. Results

### 3.1. Phantom Visualization Accuracy

The quantitative results of the phantom experiments are summarized in Table 1. TVE was assessed at three predefined target points for each of the six planned craniotomies and reported both in image space (TVE_2_D, pixels) and after conversion to millimeters (TVE_3_D, mm).

Overall, five of the six craniotomies demonstrated relatively consistent visualization accuracy, whereas Craniotomy 1 showed substantially greater error. The mean TVE_3_D for Craniotomy 1 was 6.92 ± 0.54 mm, with a maximum error of 7.83 mm. In comparison, the mean TVE_3_D for the remaining craniotomies ranged from 1.33 to 1.91 mm, with maximum errors ranging from 2.15 to 2.84 mm. The corresponding mean TVE_2_D for Craniotomies 2–6 ranged from 4.79 to 6.83 pixels, compared with 28.07 pixels for Craniotomy 1. The maximum TVE_2_D for Craniotomy 1 was 31.73 pixels.

Craniotomy 1 was affected by incorrect assembly of Phantom 1 due to undercut-related difficulties, which may have altered the positioning of the patient-specific registration template. In addition, Craniotomy 1 had a relatively large distance between the centroid of the tracking-marker configuration and the centroid of the planned trajectory (74.5 mm). This was the largest centroid distance among the six craniotomies.

As a secondary descriptive summary, when Craniotomies 2–6 were considered separately from the procedure affected by the documented phantom-assembly problem, the mean TVE_3_D across these five craniotomies was 1.64 mm.

### 3.2. AR-Guided Trajectory Tracing Performance in the Phantom Study

The ability of the operator to reproduce the pre-planned craniotomy trajectory under AR guidance was assessed by comparing the trajectory traced on the physical phantom with the corresponding virtual trajectory. Four of the six traced trajectories were completely contained within the virtual trajectory band, corresponding to 100% overlap. Partial overlap was observed for Craniotomies 2 and 3, with overlap values of 62.5% and 67.0%, respectively. For the portions of these trajectories extending beyond the virtual band, the mean deviation was 0.79 mm in both cases (Table 2).

Overall, the mean trajectory overlap across the six craniotomies was 88.2%. In both trajectories showing incomplete overlap, the mean deviation of the portions outside the virtual band remained below 1 mm.

### 3.3. Cadaveric Evaluation

**Figure 4:**
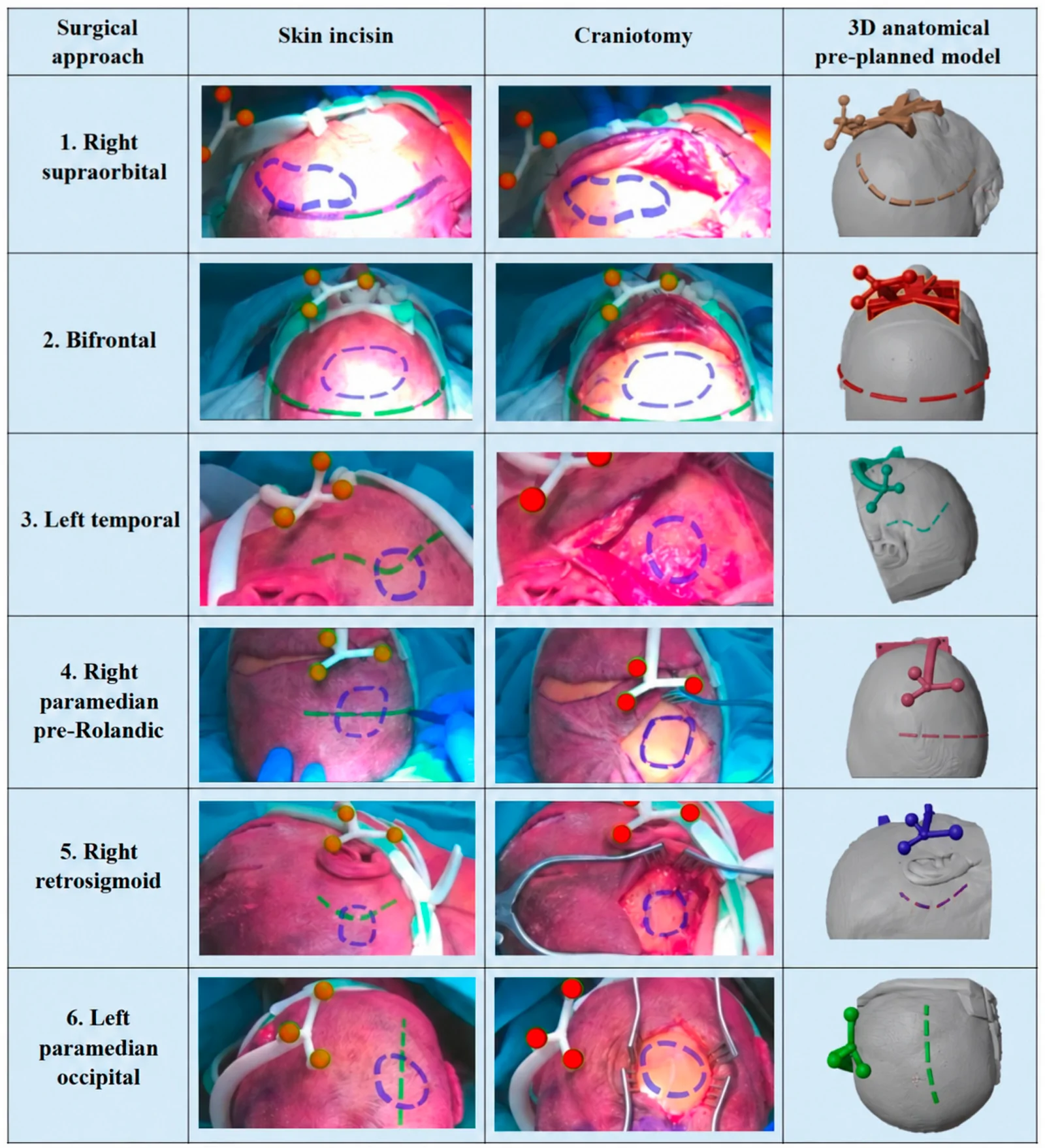
AR-guided execution of the six cranial approaches proposed. For each approach, the planned skin incision (in green), craniotomy trajectory (in blue) and corresponding patient-specific preoperative 3D model are shown.

The complete AR-guided workflow was completed for all six pre-planned craniotomies on two fresh-frozen human cadaver heads. The procedures included a right supraorbital, bifrontal, left temporal, right paramedian pre-rolandic, right retrosigmoid and left paramedian occipital craniotomy (Figure 4).

The patient-specific facial registration templates did not substantially interfere with the surgeons’ movements. The fixation system was generally effective, although additional surgical tape was applied in selected cases to further secure the registration template to the facial surface.

The correspondence between the AR-traced and planned craniotomy trajectories varied among the six procedures. Complete containment of the AR-traced trajectory within the inspection window was observed for Craniotomies 2 and 3, corresponding to the nominal ±1.5-mm centerline tolerance. For Craniotomy 3, the procedure was performed after the initial thawing-related deformation had subsided, resulting in a more stable fit of the facial registration template.

Partial containment was observed for Craniotomies 4 and 5. For Craniotomy 4, the deviation appeared to be associated with slight mispositioning of the patient-specific facial registration template.

For Craniotomy 6, only a minimal portion of the traced trajectory was contained within the inspection window. This result was associated with incorrect positioning of the facial registration template. In addition, the specimen was positioned with the face resting on the operating surface, which may have exerted pressure on the template arm and further affected its fit.

Craniotomy 1 demonstrated the lowest correspondence: the traced trajectory did not enter the inspection window at any point. Deformation of the anatomical surface during thawing altered the facial geometry relative to the CT acquisition conditions, resulting in an inadequate fit of the patient-specific registration template.

Overall, two of the six AR-traced craniotomy trajectories (33.3%) met the predefined complete-containment criterion within the inspection windows corresponding to the nominal ±1.5-mm tolerance. Of the remaining four trajectories, two were partially contained, one was minimally contained and one was not contained at any point. Incomplete containment was observed in association with thawing-related changes in facial geometry and variability in the positioning or stability of the patient-specific facial registration template.

## 4. Discussion

The present exploratory preclinical study evaluated a patient-specific AR workflow designed to assist the neurosurgeon in reproducing preoperatively planned skin-incision and craniotomy trajectories on physical cranial anatomy. Using the VOSTARS HMD-based AR platform, six different cranial approaches were planned and subsequently reproduced on patient-specific phantom models and fresh-frozen cadaveric specimens.

The principal findings were threefold. First, phantom experiments demonstrated that five of the six craniotomies showed mean TVE_3_D values ranging from 1.33 to 1.91 mm, whereas Craniotomy 1 showed a substantially higher mean TVE_3_D of 6.92 mm in association with a documented phantom-assembly problem. Second, four of the six craniotomy trajectories traced under AR guidance showed 100% overlap with the corresponding virtual trajectory; in the remaining two, the portions outside the virtual band showed a mean deviation of 0.79 mm. Third, the complete workflow was completed for all six planned approaches on two human cadaveric heads. Inspection-template assessment showed complete containment of the AR-traced trajectory within the predefined ±1.5-mm tolerance in two craniotomies; the remaining trajectories showed partial, minimal, or no containment.

The difference between phantom and cadaveric performance is an important finding of the present study. Under controlled phantom conditions, registration geometry and template positioning could be maintained more reproducibly, whereas the cadaveric setting introduced additional variability related to soft-tissue deformation, anatomical changes associated with thawing, which will not be present in real patients, template positioning and fixation stability. These factors directly affect the spatial relationship between the patient-specific template and the underlying cranial anatomy. Accordingly, the reduced performance observed in the cadaveric experiments should not be regarded solely as an experimental artefact, but may also reflect a limitation of the current template-based registration paradigm, which depends on accurate and reproducible template-to-anatomy coupling. In this context, the cadaveric model provided an essential translational step by exposing sources of error that cannot be fully reproduced in rigid phantom models and by allowing evaluation of the complete surgical workflow under anatomically realistic conditions.

More broadly, cadaveric models remain essential resources for the translational evaluation of emerging surgical technologies, while body donation continues to play a fundamental role in anatomical education and research. They provide anatomically realistic conditions in which device usability, procedural constraints, tissue-related variability, and workflow-specific sources of error can be assessed before clinical application.

Anatomical variations represent a significant challenge in AR-assisted neurosurgery and they should be considered during the development, validation, and clinical implementation of AR-based neurosurgical platforms. AR training programs should expose trainees to a wide spectrum of anatomical configurations, including uncommon but clinically significant variants, to improve their adaptability and decision-making under challenging operative conditions.

Without adequate, long-term training on cadavers, surgeons may be less prepared to recognize and adapt to these variations, potentially increasing the risk of misinterpretations and surgical complications [18,19,20]. Training on cadavers that incorporates significant anatomical variability is therefore essential to develop both manual dexterity and the ability to identify unexpected anatomical structures. Variations in arterial or neural anatomy can significantly impact preoperative planning, including craniotomy design and surgical corridor selection, and are critical to preventing surgical complications [21,22,23]. Integrating anatomical variants into AR training scenarios and validating these systems on anatomically diverse cadaveric specimens could improve their reliability and better prepare surgeons for the complexities of clinical practice.

Future investigations should examine whether anatomical variability affects the accuracy of AR registration, the reliability of anatomical landmark identification and the transfer of skills from simulation to clinical practice. In the context of increasingly digital and technology-assisted surgical training, body donor dissection therefore continues to provide a unique bridge between controlled laboratory testing and real-world surgical anatomy [24,25,26].

### 4.1. Clinical Relevance and Potential Role Alongside Conventional Neuronavigation

The clinical relevance of the proposed workflow lies in its application to the initial stages of cranial surgery, particularly the reproduction of preoperatively planned skin-incision and craniotomy trajectories on the patient’s anatomy. This may be particularly relevant in minimally invasive or anatomically constrained approaches, in which the location and geometry of the bone opening influence surgical exposure and the intended instrument trajectory.

Previous studies have investigated AR for intracranial visualization, anatomical orientation and surgical guidance [3,5,10,11]. Davidovic et al. [16] reported smaller target-oriented craniotomies with AR than with conventional neuronavigation in a simulated intracranial aneurysm procedure. Although this finding cannot be directly extrapolated to clinical practice, it supports further evaluation of AR for craniotomy planning and positioning. Previous experimental work has also specifically explored AR-assisted craniotomy planning and execution [13,27].

However, AR does not eliminate the fundamental limitations of image-guided surgery. Its performance remains dependent on accurate registration, stable tracking and reliable alignment between virtual information and patient anatomy. Depth-perception limitations, visual clutter, latency, restricted field of view and operator-related perceptual factors may also affect its use [9,12,14].

The present study extends this task-specific evaluation across six different cranial approaches. However, each approach was assessed only once and the study was not designed to compare performance among approaches. Moreover, the accuracy of the skin-incision trajectories was not quantitatively assessed. Accordingly, the potential clinical role of VOSTARS should be evaluated as part of an integrated navigation workflow rather than as a replacement for established navigation technologies.

### 4.2. Phantom Accuracy and Registration-Template Geometry

The phantom experiments demonstrated a mean TVE_3_D of 1.64 mm across Craniotomies 2–6. These results are within the same millimetric range as values reported for existing AR neuronavigation systems. Fick et al. [2], in a systematic review and meta-analysis, reported a mean target registration error of 2.5 mm for AR neuronavigation compared with 2.6 mm for conventional infrared neuronavigation.

However, TVE_3_D in the present study was derived from image-space measurements converted from pixels to millimeters and should therefore not be interpreted as equivalent to a conventional three-dimensional target registration error. This distinction is consistent with Kos et al. [12], who emphasized that AR evaluation metrics should be clearly defined and selected according to the intended application and risk profile. Direct numerical comparison should therefore be made cautiously.

The substantially greater visualization error observed for Craniotomy 1 was attributed to incorrect assembly of Phantom 1, which affected the positioning of the patient-specific registration template. The relatively large distance between the centroid of the tracking-marker configuration and the centroid of the planned trajectory in this case (74.5 mm) may also have increased sensitivity to template-positioning errors. Although the present study does not establish a causal relationship, this finding highlights the importance of template fit and of the spatial relationship between the tracking-marker configuration and the surgical target. More generally, AR accuracy depends not only on display technology but also on the registration method, tracking configuration and stability of the patient reference [2,7,12,28].

The spatial distribution of the six craniotomies was therefore a deliberate element of the study design. Although the marker arms were adapted to keep the tracking markers near each operative region, the anatomical constraint continued to derive from the facial support. A small translational or angular misalignment at this interface may propagate to the registered model and result in a larger positional deviation at a more remote surgical target. The current dataset does not permit a formal correlation between target distance and error; rather, it provides an exploratory assessment of the robustness of the same registration concept across distinct cranial regions. The centroid distance reported in Table 1 characterizes marker-to-target geometry and should not be interpreted as a direct measurement of the distance from the facial support to the surgical target.

Trajectory-tracing assessment provided a complementary task-level measure of the phantom workflow. Four of the six traced trajectories showed 100% overlap with the corresponding virtual band. For the remaining two trajectories, overlap was 62.5% and 67.0%, while the mean deviation of the portions extending outside the virtual band was 0.79 mm in both cases. Percentage overlap and spatial deviation describe different aspects of performance: overlap represents the proportion of the traced line contained within the predefined virtual band, whereas deviation quantifies the displacement of the portions extending beyond it. These findings show that incomplete trajectory overlap did not necessarily correspond to a large spatial deviation from the planned trajectory.

These results are broadly consistent with the previous VOSTARS study by Condino et al. [13], which reported a mean target visualization error of 1.3 ± 0.6 mm and 97% of the traced trajectory within 1.5 mm of the planned trajectory. Nevertheless, differences in experimental configuration and assessment methodology limit direct numerical comparison between the two studies.

### 4.3. Cadaveric Evaluation and Registration Challenges

The transition from phantom models to fresh-frozen human heads represents an important step toward assessing the feasibility of the workflow in a more anatomically realistic setting. A rigid phantom provides a controlled environment in which visualization and registration accuracy can be assessed, whereas a cadaveric head introduces tissue deformability and mechanical interaction between the registration template and the anatomical surface. The complete VOSTARS workflow was performed for all six planned cranial approaches on the two cadaveric specimens, supporting its technical feasibility across different anatomical regions.

Inspection-template assessment showed that two of the six AR-traced craniotomy trajectories were completely contained within the window corresponding to the predefined nominal ±1.5-mm tolerance. Of the remaining four trajectories, two were partially contained, one was minimally contained and one was not contained within the inspection window. As numerical containment percentages and deviations were not available for the cadaveric phase, these findings were reported descriptively and should be interpreted qualitatively. The cadaveric findings highlight the importance of maintaining an accurate and stable relationship between the patient-specific registration template and the anatomical surface. The phantom and cadaveric findings should be interpreted as complementary rather than as directly comparable accuracy rates. Four of six phantom trajectories were completely contained within the 2-mm virtual band, whereas two of six cadaveric trajectories were completely contained within the 4-mm inspection windows. Because these assessments used different tolerance bands and measurement procedures, the proportions cannot be compared quantitatively. Nevertheless, the cadaveric results show that controlled visualization and tracing performance does not fully capture the additional uncertainty introduced by soft-tissue deformation, specimen positioning, template fixation and execution of the complete surgical workflow.

This observation is consistent with the literature, in which registration accuracy and stability are repeatedly identified as central determinants of AR performance [2,3,29,30,31,32].

The observed inaccuracies were associated primarily with suboptimal positioning or fit of the patient-specific facial registration template. In one specimen, anatomical-surface deformation during thawing affected template fit, while template positioning was identified as a possible source of deviation in other approaches. These observations indicate that template fit and stability should be considered integral components of the overall AR-guided workflow.

### 4.4. Limitations and Future Neurosurgical Applications

This study has several limitations. First, only two cadaveric heads and six cranial approaches were evaluated, with each approach assessed once. The study was therefore not designed to compare accuracy among different approaches or to characterize repeatability and reproducibility. Second, no direct comparison with conventional neuronavigation was performed. Consequently, the present findings do not demonstrate an accuracy or clinical advantage over established navigation systems.

Third, the metric reported as TVE_3_D in the phantom experiments was derived from image-space measurements converted from pixels to millimeters and should not be interpreted as equivalent to a conventional three-dimensional target registration error. Moreover, the cadaveric evaluation was based on qualitative classification of trajectory containment within the inspection window because numerical containment percentages and deviations were not available. The accuracy of the planned skin-incision trajectories was not quantitatively assessed.

Fourth, phantom and cadaveric models cannot fully reproduce clinical surgical conditions. In particular, the effects of patient positioning, scalp preparation and displacement, surgical manipulation and changes in the relationship between the registration template and the anatomical surface require further investigation. The ±1.5-mm tolerance used for the inspection-template assessment should also be regarded as a study-specific experimental criterion rather than a universally accepted clinical threshold. Finally, although the six locations were selected to span different spatial relationships to the facial support, the support-to-target distance was not quantified as an independent variable and the present sample does not permit inference of a distance-error relationship. Accordingly, the cadaveric findings should be interpreted primarily as a feasibility assessment of the complete workflow rather than as a quantitative validation of surgical accuracy.

Future studies should include larger samples, repeated measurements for each cranial approach and quantitative assessment of registration stability over time. The contribution of template positioning, fixation, marker configuration and distance between the registration reference and the surgical target should be evaluated systematically. In parallel, future development will investigate markerless patient-to-image registration strategies aimed at reducing dependence on the positioning and stability of a physical facial template. Regardless of the registration strategy adopted, the workflow should incorporate an intraoperative registration sanity check to verify alignment and detect possible registration drift during the procedure [33]. Subsequent comparative studies against conventional neuronavigation will be required to determine whether the proposed workflow provides measurable advantages in craniotomy positioning, procedure time, surgeon workload, or extent of bone removal.

## 5. Conclusions

The findings support the potential of VOSTARS as a tool for patient-specific cranial surgical planning and intraoperative guidance, enabling direct transfer of preoperative skin-incision and craniotomy trajectories to the operative field. The phantom and cadaveric experiments demonstrated the technical feasibility of the workflow while also identifying limitations related to registration-template fit and stability under more realistic anatomical conditions. Given the limited number of specimens and the single execution of each approach, these findings should be interpreted as a technical feasibility assessment rather than as a formal validation of clinical accuracy.

These findings suggest that the potential clinical relevance of AR may lie not only in three-dimensional visualization but also in its ability to connect patient-specific preoperative planning with the surgical field. Future work will investigate markerless patient-to-image registration strategies to reduce dependence on the positioning and stability of a physical facial template. Further validation using larger samples, repeated measurements and direct comparison with conventional neuronavigation is required before clinical implementation.

## Figures and Tables

**Table 1:** TVE in the phantom study. Values are reported as mean, maximum and standard deviation (SD). Centroid distance represents the distance between the centroid of the tracking-marker configuration and the centroid of the planned craniotomy trajectory.

Metric	Craniotomy 1	Craniotomy 2	Craniotomy 3	Craniotomy 4	Craniotomy 5	Craniotomy 6
TVE_2_D mean (px)	28.07	5.58	6.48	6.83	4.79	5.19
TVE_2_D max (px)	31.73	8.41	9.18	9.70	8.89	8.35
TVE_2_D SD (px)	2.17	1.64	1.84	1.66	1.84	1.62
TVE_3_D mean (mm)	6.92	1.80	1.64	1.91	1.53	1.33
TVE_3_D max (mm)	7.83	2.72	2.32	2.71	2.84	2.15
TVE_3_D SD (mm)	0.54	0.53	0.46	0.46	0.90	0.42
Centroid distance (mm)	74.5	65.0	63.5	45.0	64.0	66.5

px, pixels.

**Table 2:** AR-guided trajectory-tracing performance in the phantom study.

Craniotomy	Overlap (%)	Deviation (px)	Deviation (mm)
1	100	—	—
2	62.5	2.44	0.79
3	67.0	3.16	0.79
4	100	—	—
5	100	—	—
6	100	—	—

Deviation values refer only to the portions of the traced line extending outside the virtual trajectory band. —, not applicable because the traced trajectory was completely contained within the virtual band; px, pixels.

## Data Availability

The data presented in this study are available from the corresponding author upon reasonable request and subject to institutional and ethical restrictions regarding patient privacy.
